# New therapeutic opportunities from dissecting the pre-B leukemia bone marrow microenvironment

**DOI:** 10.1038/s41375-018-0144-7

**Published:** 2018-05-08

**Authors:** Laurence C. Cheung, Jennifer Tickner, Anastasia M. Hughes, Patrycja Skut, Meegan Howlett, Bree Foley, Joyce Oommen, Julia E. Wells, Bo He, Sajla Singh, Grace-Alyssa Chua, Jette Ford, Charles G. Mullighan, Rishi S. Kotecha, Ursula R. Kees

**Affiliations:** 1 Telethon Kids Cancer Centre, Telethon Kids Institute, University of Western Australia, Perth, WA Australia; 2 School of Pharmacy and Biomedical Sciences, Curtin University, Perth, WA Australia; 3 School of Pathology and Laboratory Medicine, University of Western Australia, Perth, WA Australia; 4 Harry Perkins Institute of Medical Research, Centre for Medical Research, University of Western Australia, Perth, WA Australia; 5 Department of Pathology, St. Jude Children’s Research Hospital, Memphis, TN USA; 6 Department of Haematology and Oncology, Princess Margaret Hospital for Children, Perth, WA Australia; 7 School of Medicine, University of Western Australia, Perth, WA Australia

## Abstract

The microenvironments of leukemia and cancer are critical for multiple stages of malignancies, and they are an attractive therapeutic target. While skeletal abnormalities are commonly seen in children with acute lymphoblastic leukemia (ALL) prior to initiating osteotoxic therapy, little is known about the alterations to the bone marrow microenvironment during leukemogenesis. Therefore, in this study, we focused on the development of precursor-B cell ALL (pre-B ALL) in an immunocompetent BCR-ABL1^+^ model. Here we show that hematopoiesis was perturbed, B lymphopoiesis was impaired, collagen production was reduced, and the number of osteoblastic cells was decreased in the bone marrow microenvironment. As previously found in children with ALL, the leukemia-bearing mice exhibited severe bone loss during leukemogenesis. Leukemia cells produced high levels of receptor activator of nuclear factor κB ligand (RANKL), sufficient to cause osteoclast-mediated bone resorption. In vivo administration of zoledronic acid rescued leukemia-induced bone loss, reduced disease burden and prolonged survival in leukemia-bearing mice. Taken together, we provide evidence that targeting leukemia-induced bone loss is a therapeutic strategy for pre-B ALL.

## Introduction

Acute lymphoblastic leukemia (ALL) is the most common cancer among children and remains a frequent cause of death from cancer before 20 years of age [1, 2]. Survival for children and adolescents with ALL has greatly improved over recent decades, with long-term survival now exceeding 85%, primarily due to combination therapies, improved supportive care, and the introduction of novel agents such as tyrosine-kinase inhibitors [1–6]. A significant gain in clinical outcome has been achieved through better prediction of survival, based on refined risk stratification of patients. The detection of minimal residual disease is the single most powerful predictor, and is critical in selecting optimal therapy for each patient [1, 4, 6]. However, outcomes in high-risk subgroups and salvage rates remain poor, including those with BCR-ABL1 fusion, BCR-ABL1-like ALL, T-cell ALL (T-ALL), and infant ALL [1, 5, 7–9]. Further intensification of current multi-agent chemotherapy is associated with increased toxicity, and hematopoietic stem cell transplantation is an option for patients who are considered to be at very high risk of treatment failure. Hence, finding less toxic and more effective therapies for high-risk ALL subgroups is vital.

Advances in immunological approaches have led to the development of novel therapies for immune checkpoint blockade and the targeting of surface antigens on leukemic cells. Genetically modified antibodies directed at CD19, CD20, CD22 and CD30 antigens on hematopoietic tumors have been reported to demonstrate anti-leukemic activity as single agents [10–13]. Initial chimeric antigen receptor T-cell therapies were developed to primarily target the CD19 cell surface antigen that is present at high density on most precursor-B cell ALL (pre-B ALL). In pioneering clinical trials, potent effects have been demonstrated in relapsed and refractory pre-B ALL [11, 14, 15]. Immunological approaches have the capacity to overcome chemotherapy resistance.

Another novel therapeutic approach is targeting the microenvironment of hematopoietic tumors [16, 17]. The role of the bone marrow microenvironment (BMM) in driving disease progression is widely recognized, with chemokine receptors (CXCR4), adhesion molecules, signal transduction pathways and hypoxia-related proteins playing a role [18–26]. The recent recognition that the tumor microenvironment contributes to treatment failure or success has highlighted the need to improve our understanding of the signaling programs elaborated by the microenvironment [27, 28]. Could existing cancer therapies be improved by the addition of novel therapies directed at signaling programs? It is well documented that malignant cells have the capacity to remodel the BMM, thereby promoting disease development [22, 23, 25, 26, 29–34]. To identify novel targets and signaling programs, greater understanding of the complex interactions within the BMM is required. Exploiting unique properties of the leukemia microenvironment has great potential.

Pre-B ALL is the most common form of leukemia in children. Symptoms at the time of presentation include bruising, bleeding, pallor, fatigue, and infections [1]. More than 35% of patients suffer from musculoskeletal pain, and skeletal abnormalities are frequently present at diagnosis [35]. Low serum markers of bone formation have been recorded prior to commencing therapy, and bone histomorphometric assessment has identified a reduction in trabecular bone volume as well as trabecular thickness [35–37]. Bone marrow trephines at diagnosis of pediatric ALL show lower percentages of adipocytes, osteoblasts and osteoclasts, strongly suggesting that ALL cells have the capacity to alter the BMM [38]. However, their precise impact on hematopoiesis, bones and BMM remain obscure. To elucidate the impact of leukemia development in pre-B ALL, we generated and investigated a novel immunocompetent BCR-ABL1^+^ model.

## Methods

### Retroviral Production

MSCV vector coexpressing human BCR-ABL1 (p185) and mCherry (MSCV-BCR-ABL1-IRES-mCherry) was obtained from St Jude Children’s Research Hospital, Memphis, TN, USA and has been previously described [39]. 293T cells were transfected with pMD-old-gag-pol, pCAG-Eco and MSCV-BCR-ABL1-IRES-mCherry using FuGENE 6 (Promega, Madison, WI, USA). At 48 h after transfection, viral supernatants were collected, filtered, and stored at −80 °C.

### Mouse Modeling of BCR-ABL1 pre-B Leukemia

**Fig. 1:**
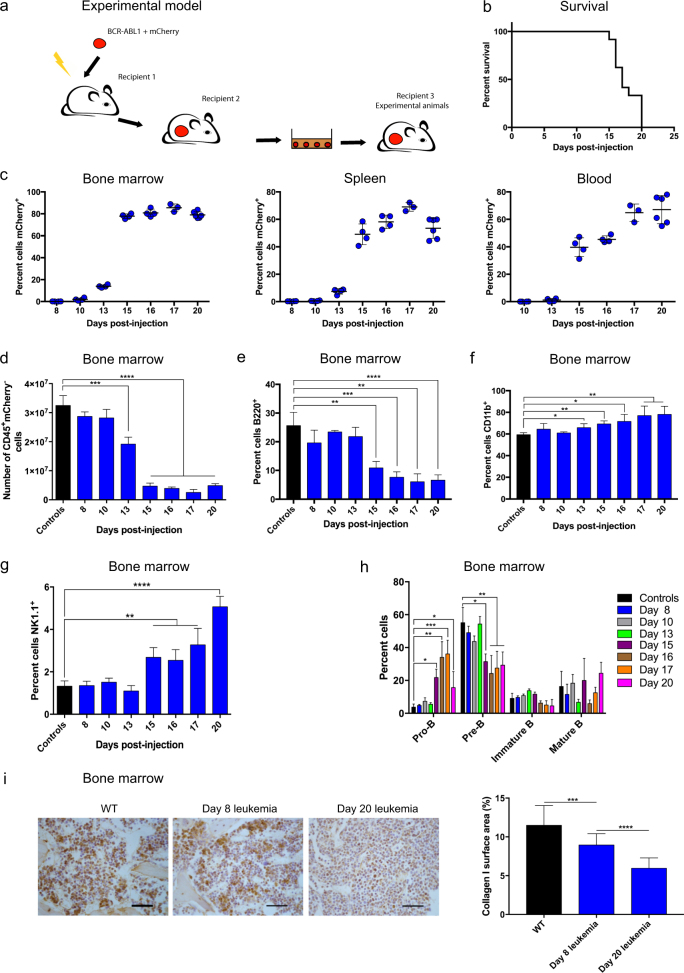
Pre-B leukemia impairs hematopoiesis in the bone marrow. **a** Schematic representation of the experimental model. The normal bone marrow cells were transduced with MSCV-BCR-ABL1-IRES-mCherry retrovirus and transplanted into lethally irradiated Recipient 1. All subsequent transplantations were performed using non-irradiated recipients. **b** Kaplan–Meier survival curve of mice transplanted with 1000 *BCR-ABL1* ALL cells (*n* = 24 mice). **c** Percentage of mCherry^+^ cells in bone marrow, spleen and blood during leukemia development. **d** Number of hematopoietic cells (CD45^+^mCherry^−^) in the bone marrow during leukemia development. **e** Percentage of B220^+^ cells in the CD45^+^mCherry^−^ fraction during leukemia development. **f** Percentage of CD11b^+^ cells in the CD45^+^mCherry^-^ fraction during leukemia development. **g** Percentage of NK1.1^+^ cells in the CD45^+^mCherry^-^ fraction during leukemia development. **h** Composition of B cell subpopulations during leukemia development (percentage in the B220^+^CD45^+^mCherry^-^ fraction). **c-h** Bone marrow cells were harvested from one femur and two tibias (Day 8, 10, 13, 15, and 16: *n* = 4 mice; Day 17: *n* *=* 3 mice; Day 20: *n* = 6 mice). **i** Representative images of immunostaining for collagen I (brown) from WT and mice at Day 8 and Day 20 post leukemia cell injection (scale bars, 50 μm) (left) and quantification of the percentage of collagen I per surface area (*n* = 3) (right). Throughout, *P < 0.05, **P < 0.01, ***P < 0.001, ****P < 0.0001. Error bars represented mean ± SD

Seven to 10-week old C57BL/6J mice were purchased from the Animal Research Centre, Perth. Animals were housed under pathogen-free conditions and all studies were approved by the Animal Ethics Committee, Telethon Kids Institute, Perth. Bone marrow cells from C57BL/6 J mice were extracted from femurs and tibias and transduced with MSCV-BCR-ABL1-IRES-mCherry retroviral supernatant supplemented with 100 ng/mL stem cell factor, 10 ng/mL IL-6, 50 ng/mL thrombopoietin, and 5 ng/mL Flt3 ligand (R&D Systems, Minneapolis, MN, USA) at 2500 rpm for 2 h at room temperature. One million unsorted cells were transplanted into lethally irradiated (two doses of 550 cGy with a 2 h interval between doses) recipients (Recipient 1) via tail vein injections (Fig. 1a). Irradiation of recipient mice was not required in the secondary transplantation of leukemia cells (Recipient 2). Leukemia cells harvested from Recipient 2 were expanded and maintained in RPMI supplemented with 10% fetal calf serum, penicillin–streptomycin (Thermo Fisher Scientific, Waltham, MA, USA), glutamine and 55 μM 2-mercaptoethanol. All experiments were conducted using tertiary transplantation recipients (Recipient 3). Each recipient received 1000 leukemia cells, except for the homing study where 10^6^ cells were transplanted.

### Flow Cytometry and Cell Sorting (FACS)

All FACS studies were performed using single cell suspensions, and cells were stained using standard protocols. Flow cytometry was performed on a BD Fortessa and FACS on a FACSAria. Cell suspensions were counted using a Vi-CELL® Cell Viability Analyzer (Beckman Coulter, Indianapolis, Indiana, USA). BD Horizon^TM^ Fixable Viability Stain 700 (BD Biosciences, Franklin Lakes, NJ, USA) was used for exclusion of dead cells. For phenotypic analysis of mouse bone marrow hematopoietic cells, mononucleated cells were treated with Red Blood Cell Lysis Buffer (Thermo Fisher Scientific) and stained with CD45−FITC, B220-PerCP-Cy5.5, CD19−APC-H7, CD43-BV421, IgM-PE-Cy7, IgD-BV786, CD11b-BV605, Ly6G-APC-H7, Ly6C-BV421, F4/80-PE, CD3-BV510, and NK1.1-BV786. For phenotypic analysis of leukemia cells, CD19-APC-H7, CD24-APC, and BP-1-PE were used. For phenotypic analysis of bone marrow stromal cells, femurs and tibias were crushed and incubated with 337.5 U/mL collagenase (Worthington Biochemical Corp., Lakewood, NJ, USA) and 40 U/mL DNase I (Sigma-Aldrich, NSW, Australia) at 37 °C for 60 min in a shaking water bath. Digested bone fragments were filtered through sterile 100 μm strainers. Mononucleated cells were treated with Red Blood Cell Lysis Buffer and stained with CD45-PerCP-Cy5.5 and Ter119-PerCP-Cy5.5 for exclusion of hematopoietic cells. Subtypes of bone marrow stromal cell were identified using Sca-1-BV510, CD31-FITC, PDGRFα-APC, PDGFRβ-PE, and CD51-biotin/streptavidin-BV421. To enumerate bone marrow fibroblasts, cells were fixed, permeabilized, and stained with vimentin-APC (R&D Systems). For purification of bone marrow stromal cells, CD45-PerCP-Cy5.5 and Ter119-PerCP-Cy5.5 were used to exclude the hematopoietic cells. For purification of pre-B cells, CD19-APC-H7, CD24-APC, BP-1-PE, and IgM-PE-Cy7 were used.

### Tissue Processing and Immunohistochemistry

For histological analysis, femurs were fixed in 4% paraformaldehyde (Sigma Aldrich) in PBS at 4 °C for 48 h and decalcified in 10% EDTA at 4 °C for 8 days. Tissues were embedded in paraffin and were cut at 5 μm thick for immunohistochemistry, hematoxylin and eosin staining, or tartrate-resistant acid phosphatase (TRAP) staining. The slides were first incubated at 60 °C for 45 min and then deparaffinized using a Leica Autostainer XL (Leica microsystems, Wetzlar, Germany). For TRAP staining, slides were immersed in pre-warmed TRAP staining solution (50 mM sodium acetate (pH 5.2), 0.15% Naphthol-AS-TR-phosphate, 50 mM sodium tartrate, and 0.1% Fast Red TR) at 37 °C in the dark for 30 min and were counterstained with hematoxylin (Vector Labs, Burlingame, CA, USA). Images were obtained using Pannoramic MIDI (3DHistech Ltd, Budapest, Hungary). Bone histomorphometry parameters were then analyzed using BIOQUANT OSTEO software (BIOQUANT Image Analysis Corporation, Nashville, TN, USA). For collagen staining, deparaffinized femoral sections were stained with collagen type I antibody (Abcam, Cambridge, MA, USA) overnight at 4 °C followed by biotinylated goat anti-rabbit IgG (VectorLabs, Burlingame, CA, USA). The slides were included with avidin–biotin–peroxidase complex (VectorLabs) and were counterstained with hematoxylin. Images were obtained using a Nikon Ti-E microscope and were analyzed by NIS software modules (Nikon Instruments Inc., Melville, NY, USA).

### Microcomputed Tomography (Micro-CT) Analysis

Fixed femoral bone samples were immobilized in a 2-mL tube filled with PBS for micro-CT scanning. Samples were loaded into the bed of a Skyscan 1176 micro-CT scanner (Bruker, Kontich, Belgium) and the distal femur from midshaft to the distal articular surface was scanned using the following parameters: 50 kV, 500 µA, 1000 ms, 0.5 mm Al filter, 9-µm pixel resolution, rotation step of 0.4°, frame averaging of 2. Scans were reconstructed using NRecon software (Bruker) at a constant threshold value, and then orientated for analysis using DataViewer software (Bruker). Trabecular bone parameters were analyzed within the secondary spongiosa, defined as a region beginning 0.5 mm below the bottom of the growth plate and extending 1 mm proximally, and excluding the cortical bone. Cortical bone parameters were analyzed within the mid-diaphyseal region located 4.5 mm proximal to the base of the distal growth plate and extending for 1 mm in height proximally.

### Enzyme-linked immunosorbent assay (ELISA) Analyses

Blood was collected via cardiac puncture and kept undisturbed at room temperature for 2 h. Sera were removed by centrifuging at 5500 rpm for 10 min and stored at −80 °C. Carboxy−terminal cross-linked telopeptides of type 1 collagen (CTX) ELISA was performed in duplicate according to manufacturer’s instructions (AC-06F1, Immunodiagnostic Systems, Tyne & Wear, United Kingdom). To measure receptor activator of nuclear factor κB ligand (RANKL) protein levels from the cell supernatants after 48 h culture and bone marrow supernatants after flushing the bone marrow with 500 μL of PBS, RANKL Quantikine ELISA kit (R&D Systems) was used and the assay was performed in duplicate according to manufacturer’s instructions.

### Western Blotting

Cells were harvested, sectioned and lysed in Evan’s protein lysis buffer (1 mM EDTA, 1 mM EGTA, 1%NP-40, 50 mM Tris-HCl, 120 nM NaCl and peptide inhibitors). Ten micrograms of protein extracts from each cell line were separated electrophoretically (SDS-PAGE). The membrane was blocked with 5% milk in PBS and stained with rabbit anti-mouse sRANKL antibody (Abcam) and anti-rabbit horseradish peroxidase (GE Healthcare, Pittsburg, PA, USA). Horseradish peroxidase activity was detected with a chemiluminescence detection kit. After exposure and development of the film, the membrane was stripped and re-probed for housekeeping protein mouse anti-β-Actin (Sigma-Aldrich). Images were obtained using ImageLab Software (Bio-Rad, Hercules, CA, USA).

### RNA Extraction and Quantitative Reverse Transcription PCR

Total RNA was extracted from mCherry^+^ leukemia cells, bone marrow stromal cells (CD45^-^Ter119^-^), B cells (B220^+^), and pre-B cells (CD19^+^CD24^+^BP-1^+^IgM^-^) using RNeasy Mini Kit or RNeasy Micro Kit with RNase-free DNase I (Qiagen, Hilden, Germany). cDNA was synthesized using SuperScript VILO Master Mix (Thermo Fisher Scientific). Quantitative PCR was performed on an ABI 7900HT thermocycler using Taqman Gene Expression Assays (Thermo Fisher Scientific) for mouse *Rankl* (Mm00441906_m1), *Csf1* (Mm00432686_m1), *Opg* (Mm00435454_m1), *Pax5* (Mm00435501_m1) as well as *Hprt* (Mm03024075_m1), and SYBER Green (Qiagen) with the following specific primer set: osteocalcin F: GCGCTCTGTCTCTCTGACCT, osteocalcin R: ACCTTATTGCCCTCCTGCTT, *Hprt* F: GCAGTACAGCCCCAAAATGG, *Hprt* R: AACAAAGTCTGGCCTGTATCCAA. Relative expression was calculated using the ΔΔCT method normalized to *Hprt* levels for each individual sample measured in duplicate.

### Co-culture Study

A total of 1 × 10^4^ RAW 264.7 cells were seeded in 24-well plates in triplicate and cultured for 8 days in the presence of 20 ng/mL of RANKL protein (Abcam) or 1 × 10^5^ leukemia cells with a change of medium every 2 days. The cells were fixed and TRAP staining was performed.

### In vitro Zoledronic Acid Study

A total of 1 × 10^4^ leukemia cells were seeded in 96-well plates in triplicate and cultured for 48 h in the presence of 1 μM or 10 μM of zoledronic acid. Ten microliters of alamarBlue were added and colorimetric changes were measured by Synergy Mx Microplate Reader (BioTek, Winooski, VT, USA) at wavelengths of 570 nm and 600 nm. Percentage of alamarBlue reduction was calculated using standard formula. For the colony forming assay, 1 × 10^3^ leukemia cells were seeded in 6-well plates in triplicate and cultured for 7 days in the presence of 10 μM of zoledronic acid in Mouse Methylcellulose Complete Media for Pre-B Cells (R&D Systems).

### In vivo Zoledronic Acid Treatment

Mice were given a daily intraperitoneal (ip) injection of 2 μg of zoledronic acid (Selleckchem, Houston, TX, USA) in 100 μL PBS or 100 μL of PBS alone, 5 days a week for 2 weeks. Treatment was started at 3 days post leukemia cell injection.

### Statistical Analyses

Statistical analyses and graphics were performed using Prism 7 (GraphPad, La Jolla, CA, USA) and Microsoft Excel for Mac 2011. Data were analyzed using the two-tailed unpaired Student’s *t*-test. Survival studies were analyzed using log-rank test. The results are presented as means ± standard deviation (SD). A *P* value < 0.05 was considered as statistically significant. No statistical test was used to determine the sample size. No randomization was used in animal studies. The survival studies were blinded.

## Results

### An immunocompetent murine model of pre-B ALL

We developed and characterized an immunocompetent BCR-ABL1^+^ model (Fig. 1a). Normal bone marrow cells were transduced with MSCV-BCR-ABL1-IRES-mCherry retrovirus and transplanted into lethally irradiated recipients, and they developed pre-B ALL. Secondary recipients received leukemia cells without any pre-conditioning. The BCR-ABL1^+^ cells isolated from the secondary recipients were confirmed to express B-lineage markers and they could be expanded and maintained in culture (Supplementary Figure 1A) [39]. Experiments were performed using immunocompetent recipients injected with 1000 BCR-ABL1^+^ cells, thus avoiding irradiation-induced changes to the microenvironment. Leukemia cells homed to bone marrow and spleen, and mice developed BCR-ABL1^+^ leukemia within 3 weeks (Fig. 1b; Supplementary Figure 1B). Leukemia cells remained at a low level of <1% for 10 days, followed by rapid expansion in bone marrow and spleen, and slightly later in blood (Fig. 1c).

### Pre-B leukemia impairs hematopoiesis in the bone marrow

In order to detect any changes in the hematopoietic and stromal bone marrow compartments due to the presence of low levels of leukemia cells, we used a range of techniques, methodically assessing hematopoiesis, extracellular matrix (ECM) and bone marrow stroma (Supplementary Figure 2). Leukemia mice showed a reduction of normal hematopoietic cells (CD45^+^mCherry^−^) in the bone marrow (Fig. 1d) and massive splenomegaly (Supplementary Figure 1C). Further, we observed lower proportions of B cells, and higher proportions of myeloid and NK cells but not CD3^+^ T cells (Fig. 1e–g; Supplementary Figure 1D), accompanied by increased proportions of neutrophils, Ly6c^lo^ monocytes and macrophages (Supplementary Figure 1E). Starting on Day 15, we recorded a significant increase of pro-B cells and a corresponding drop in pre-B cells within the B220^+^ fraction, suggesting leukemia development impairs the pro-B to pre-B differentiation (Fig. 1h).

### Leukemia reduces collagen type I and decreases the number of endothelial and osteoblastic cells in the bone marrow

**Fig. 2:**
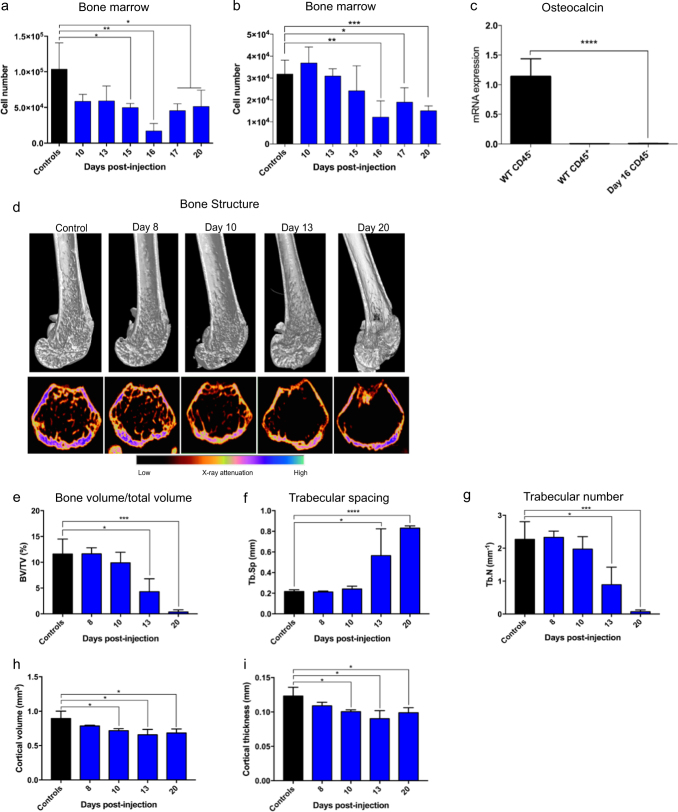
Pre-B leukemia induces bone loss in vivo. **a**, **b** Bone marrow cells were harvested from one femur and two tibias after enzymatic dissociation (Day 8, 10, 13, 15, and 16: *n* = 4 mice; Day 17: *n* *=* 3 mice; Day 20: *n* = 6 mice). **a** Number of endothelial cells. **b** Number of osteoblastic cells. **c** Mean expression of osteocalcin (*Bglap*) in bone marrow CD45^−^ cells (WT), CD45^+^ (WT) and in CD45^−^ cells in leukemia-bearing mice at Day 16 post leukemia cell injection (*n* = 3). **d** 3D rendering of distal femur bone compartment (top panels: longitudinal sections; bottom panels: axial sections). Representative cortical and trabecular bones in the distal femurs of mice at stated days post leukemia cell injection (false colored images, *n* = 4 mice per time point). **e–g** Micro-CT analysis of femoral trabecular bone (*n* = 4 mice per time point). **e** Bone volume/total volume (BV/TV). **f** Trabecular spacing (Tb.Sp). **g** Trabecular number (Tb.N). **h–i** Micro-CT analysis of femoral cortical bone at trabecular zone (*n* = 4 mice per time point). **h** Cortical volume. **i** Cortical thickness. Throughout, *P < 0.05, ** *P* < 0.01, *** *P* < 0.001, **** *P* < 0.0001. Error bars represented mean ± SD

To test the effect on the ECM and bone marrow stromal compartment, we examined the level of collagen type I by immunohistochemistry, and enumerated the subpopulations of stromal cells by flow cytometry using enzymatically digested bones. The content of collagen type I was significantly reduced in femurs at Day 8 and Day 20 post leukemia cell injection (Fig. 1i). While leukemia did not affect the number of mesenchymal stem/stromal cells (MSCs) [40], PDGFRα^+^Sca-1^+^ (PαS) mesenchymal cells [41], CXCL-12 abundant reticular cells [42] and vimentin^+^ fibroblasts [43], we observed significantly lower numbers of endothelial and osteoblastic cells (Fig. 2a, b; Supplementary Figure 3). Consistent with this, expression of osteocalcin mRNA was significantly reduced in bone marrow stromal cells from leukemia mice, as compared to wild type (WT) mice (Fig. 2c). Collectively, our results demonstrated that the presence of pre-B ALL cells perturbs hematopoiesis and alters the BMM.

### Leukemia induces bone loss

Next we investigated the effect of leukemia cells on bones. Strikingly, we found progressive bone loss over the course of the disease (Fig. 2d). Although leukemia did not affect the cortical bone in the mid-femoral diaphyseal region (Supplementary Figure 4), leukemia mice exhibited loss of trabecular structures in the metaphyseal region of distal femurs (Fig. 2e). At Day 13 and 20, trabecular spacing was significantly increased and trabecular numbers were decreased in leukemia mice (Fig. 2f, g). Micro-CT analysis also revealed severe loss of cortical bone in the same region from Day 10 onward (Fig. 2h, i). Thus, development of pre-B leukemia induces bone loss, and this model recapitulates the clinical symptoms of decreased bone mass in children diagnosed with ALL [35].

### Leukemia enhances the activity of osteoclasts

**Fig. 3:**
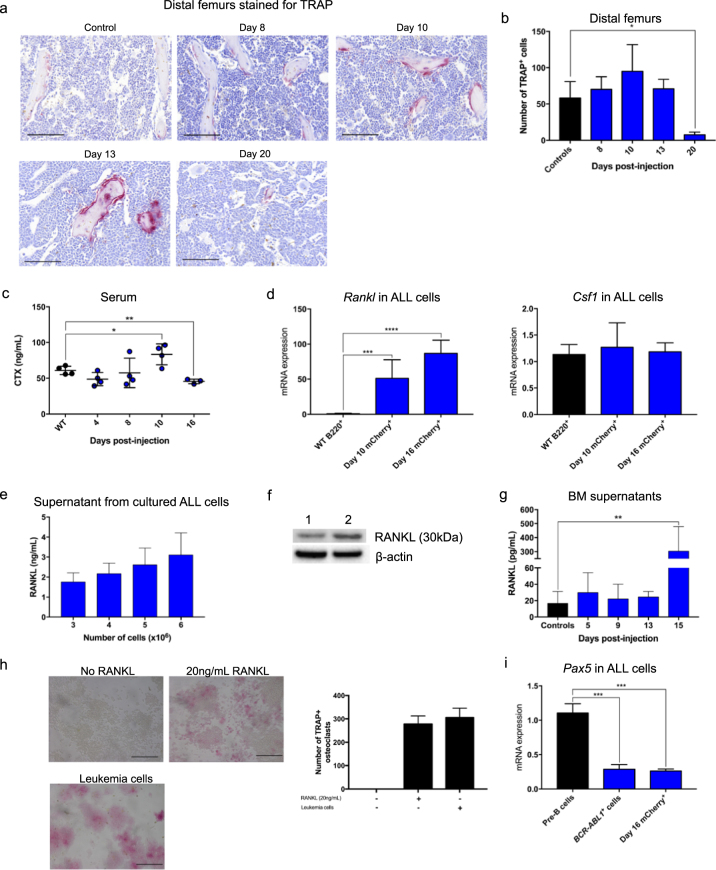
Pre-B ALL increases the activity of osteoclasts and produces high levels of RANKL. **a** Representative images of paraffin sections of distal femur bone compartment stained for the osteoclastic marker TRAP at stated days post leukemia cell injection (*n* = 4 mice per time point, scale bars, 50 μm). **b** Quantification of TRAP^+^ osteoclasts in the distal femur bone compartment. **c** Serum levels of CTX in WT and leukemia mice (WT, Day 4, 8, and 10: *n* = 4 mice; Day 16: *n* *=* 3 mice). **d** Mean expression of *Rankl* and *Csf1* in B220^+^ cells in WT mice relative to that in mCherry^+^ cells in mice at Day 10 and 16 post leukemia cell injection (WT, Day 10: *n* = 4 mice; Day 16: *n* *=* 3 mice). **e** Leukemia cells were cultured for 48 h and the level of RANKL in the supernatants was measured by ELISA (*n* = 3). **f** Representative western blot of RANKL from leukemia cell lysate (*n* = 3). Lane 1 is B220^+^ cell lysate and Lane 2 is leukemia cell lysate. β-actin was used as a loading control. **g** RANKL levels measured by ELISA in bone marrow supernatants from control and leukemia mice (*n* = 3). **h** Representative images of RAW 264.7 cells stained for TRAP after culture with 20 ng/mL RANKL or 1 × 10^5^ leukemia cells for 8 days (scale bars, 100 μm) (left) and quantification of the TRAP^+^ multinucleated osteoclasts (*n* = 3) (right). **i** Mean expression of *Pax5* in pre-B cells (CD19^+^CD24^+^BP-1^+^IgM^−^) in WT mice relative to that in BCR-ABL1^+^ cells (Fig. 1a) and in mCherry^+^ cells in mice at Day 16 post leukemia cell injection (*n* = 3). Throughout, *P < 0.05, **P < 0.01, ***P < 0.001, ****P < 0.0001. Error bars represented mean ± SD

Bone homeostasis relies on balanced activities of osteoblastic cells and osteoclasts. To determine whether the observed bone loss could be the result of increased osteoclast activity, we evaluated the osteoclast-mediated bone resorption during leukemia development. Histologic bone sections revealed high TRAP activity at Day 10 and 13, indicating the TRAP^+^ osteoclasts were highly active (Fig. 3a). The number of TRAP^+^ multinucleated osteoclasts was elevated at Day 10 (Fig. 3b), and the number of TRAP^+^ multinucleated cells per bone surface increased significantly from Day 10 onwards (Supplementary Figure 5A). On Day 20 the TRAP^+^ multinucleated osteoclasts were significantly reduced, which correlates with the loss of trabecular structure, as shown in Fig. 2d. Consistent with osteoblastic cells detected by flow analysis (Fig. 2b), the number of osteoblastic cells was reduced at the late stage of disease development (Supplementary Figures 5B and C). To evaluate the bone resorption activity during leukemia development, we measured serum levels of CTX, a marker of bone resportion, by ELISA. On Day 10 the CTX level was significantly higher compared to control (Fig. 3c), supporting the findings from TRAP measurements. At Day 16 CTX levels were significantly lower, which could be explained by reduced numbers of osteoclasts later in leukemia development (Fig. 3b).

### Leukemia cells produce RANKL and osteoclast-mediated bone resorption

Osteoclasts differentiate from myeloid precursors under the influence of RANKL, macrophage colony stimulating factor (M-CSF), and a soluble receptor antagonist RANKL, termed osteoprotegerin (OPG) [44]. To gain insight into the mechanism of osteoclast-mediated bone resorption, we evaluated mRNA expression of *Rankl (Tnfsf11)*, M-CSF (*Csf1*), and *Opg* in leukemia cells. *Opg* mRNA was undetectable in the leukemia cells and controls, and *Csf1* mRNA expression in leukemia cells was similar to controls (Fig. 3d). In contrast, expression of *Rankl* mRNA in leukemia cells was 50–100-fold higher than controls (Fig. 3d). In addition, abundant RANKL protein was detected in cell lysates and culture supernatants (Fig. 3e, f). ELISA analysis confirmed that Day 15 leukemia mice contained 20-fold higher levels of RANKL protein in the bone marrow supernatant than control mice (Fig. 3g). We further elucidated the effect of RANKL produced by leukemia cells on the osteoclasts by co-culturing the leukemia cells with the RAW 264.7 murine cell line [45]. We clearly observed that RAW 264.7 cells differentiated into TRAP^+^ osteoclasts in the presence of leukemia cells (Fig. 3h), confirming that RANKL secreted by leukemia cells is responsible for the osteoclast-mediated bone resorption. In addition, RANKL is documented to be strongly repressed by *PAX5*, which is a critical B-lymphoid transcription factor, and genetic lesions in *PAX5* are widespread in pre-B ALL, generally leading to reduced expression [46–48]. We found *Pax5* mRNA levels in Day 16 leukemia cells to be significantly lower compared to WT pre-B cells (Fig. 3i). Collectively, these findings highlight an important role for osteoclast-mediated bone loss in leukemia.

### Zoledronic acid reduces leukemia burden and improves survival in mice

**Fig. 4:**
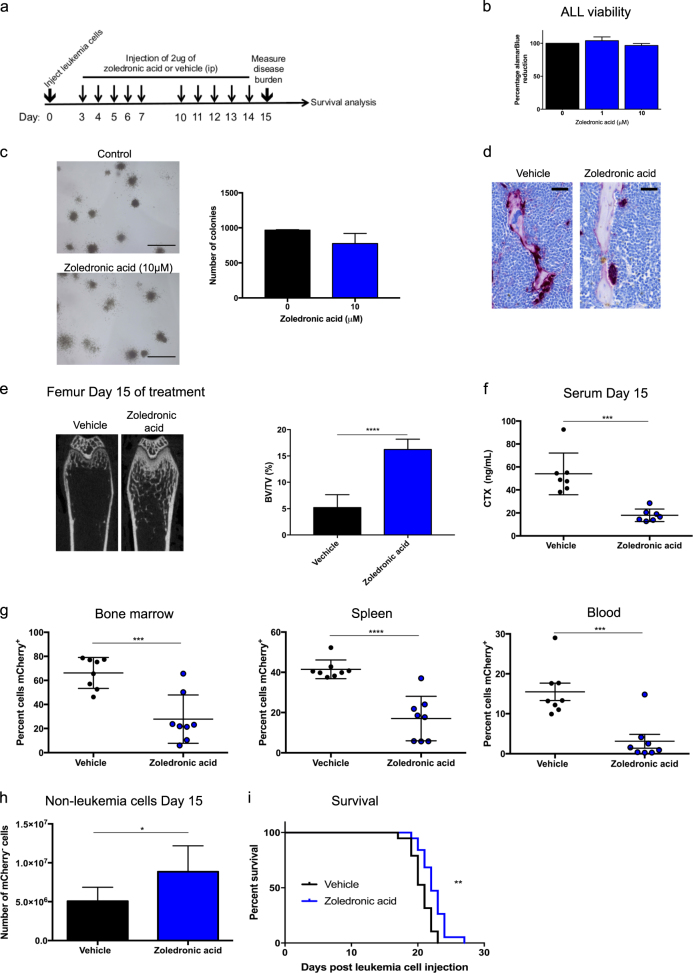
Zoledronic acid reduces leukemia burden and improves survival in mice. **a** Schematic diagram of zoledronic acid treatment schedule. **b** Leukemia cells were incubated with 0, 1 μM, or 10 μM of zoledronic acid for 48 h. Cell viability was evaluated by alamarBlue assay (*n* = 4). **c** Representative images of colony forming cells from leukemia cells treated with 0 or 10 μM of zoledronic acid for 7 days (scale bars, 500 μm) (left) and quantification of the number of colony forming cells (*n* = 3) (right). **d** Representative images of paraffin sections of distal femur bone compartment stained for the osteoclastic marker TRAP at Day 15 in mice treated with zoledronic acid or vehicle (scale bars, 10 μm). **e** Micro-CT analysis (left) and quantification (right) of femur trabecular bone at Day 15 in mice treated with zoledronic acid or vehicle (*n* = 8 mice/group). **f** Serum level of CTX at Day 15 in mice treated with either zoledronic acid or vehicle (*n* = 7 mice/group). **g** Percentages of mCherry^+^ leukemia cells at Day 15 in the bone marrow, spleen, and blood in mice treated with either zoledronic acid or vehicle (*n* = 8 mice/group). **h** Number of non-leukemia cells at Day 15 in the bone marrow of mice treated with either zoledronic acid or vehicle (*n* = 8 mice/group). **i** Kaplan–Meier survival curves of leukemia mice that were treated with either zoledronic acid or vehicle (*n* = 19 mice/group). Throughout, *P < 0.05, ***P < 0.001, ****P < 0.0001. Error bars represented mean ± SD

We next determined whether targeting the BMM could reduce the development of pre-B ALL. Given that osteoclasts are key effectors of leukemia-induced bone loss in our model, we assessed the efficacy of zoledronic acid, an osteoclast inhibitor, with treatment starting on Day 3 post leukemia cell injection (Fig. 4a). Zoledronic acid inhibited the activity of osteoclasts, restored the bone loss and reduced the bone resorption activity without direct effect on homing, colony forming capacity and viability of the leukemia cells (Fig. 4b–f; Supplementary Figure 6 and 7A). Notably, mice treated with zoledronic acid showed significantly lower leukemia burden in bone marrow, spleen and blood, as well as higher numbers of normal bone marrow cells compared to control mice at Day 15 (Fig. 4g, h). Administration of zoledronic acid prolonged survival in our aggressive leukemia model (Fig. 4i). As expected, at the time the mice succumbed to leukemia the disease burden was similar in all groups (Supplementary Figure 7B and C). These findings raise the possibility that osteoclasts may serve as therapeutic targets in pre-B ALL that is frequently associated with bone loss.

## Discussion

The main strategy for cancer therapy has focused on targeting malignant cells, leading to markedly improved cure rates for pediatric cancers. However, the recent recognition that the tumor microenvironment contributes to treatment failure or success has led to a paradigm shift [27, 28]. The BMM is essential for normal hematopoiesis, but also for leukemogenesis [26, 49, 50]. Bone marrow is a complex organ, containing many different hematopoietic and non-hematopoietic cell types that are surrounded by a shell of vascularized and innervated bone. Interactions among these cell types, secreted factors and the ECM form an intricate network that is clearly abnormal in hematopoietic malignancies.

The salient features of our pre-B ALL model are the injection of only 1000 BCR-ABL1^+^ cells into immunocompetent recipients, and without incurring any irradiation-induced damage. We observed that leukemia development perturbs hematopoiesis and impairs B-lymphopoiesis. This could be explained by leukemogenesis resulting in the altered function of the hematopoietic stem and progenitor cells [51]. Importantly, we documented severe bone loss clearly shown by Micro-CT imaging (Fig. 2d), recapitulating the clinical symptoms of decreased bone mass in children diagnosed with ALL [35]. We further investigated the bone loss, and recorded reduced numbers of osteoblastic cells and enhanced activity of osteoclasts. RANKL and CSF-1 are critical for osteoclast differentiation [44]. Mechanistically, we recorded high levels of *Rankl* mRNA but not *Csf1* mRNA in leukemia cells. We found 20-fold higher levels of RANKL protein in the bone marrow supernatant of leukemia mice compared to control mice, and the leukemia cells induced osteoclast formation in RAW 264.7 cells, demonstrated to be RANKL-dependent, documenting an important role of the RANKL signaling pathway in leukemia-induced bone loss. Our finding that ALL development severely affects key players in the BMM, including osteoblastic cells and osteoclasts, is consistent with their role in regulating the healthy hematopoietic stem cell niche and in hematopoietic tumors, including myeloid leukemia, myelodysplasia and models of T-ALL [32, 33, 52–57].

The clinical diagnostic findings in children with ALL are reflected in our model, particularly with respect to hematopoiesis, bone and mineral homeostasis. Our observation of collagen reduction during leukemia development is consistent with measurements in bone marrow fibroblasts derived from patients with ALL [58]. The presence of RANKL mRNA expression has also been reported in the majority of primary human adult and pediatric B-lineage ALL specimens [59], providing further clinical corroboration of our model.

Successful establishment of a leukemia in the bone marrow involves the co-evolution of the malignant cells, immune and stromal cells, and the ECM. The findings from this study show that the non-transformed cell types and the ECM of the bone marrow are modified early in the development of leukemia, most likely via interactions that are mediated through direct cell contact and secreted cytokines, chemokines and other factors. Novel insights for hematopoietic tumors originated from targeting the microenvironment of myeloid neoplasms. Single-cell transcriptomics has revealed deregulation of stromal cells in patients with chronic myeloid leukemia [60]. In murine models of myelodysplastic syndrome and acute myeloid leukemia, normalization of the BMM as well as reinstatement of osteoblast number and function led to altered disease progression and prolonged survival [52, 61]. The BMM not only acts as a sanctuary for leukemia cells but is also a fertile soil for cancer metastasis [62]. Tumor invasion into bone is associated with RANKL-mediated osteoclastogenesis [63].

Our preclinical model recapitulates the clinical symptoms of bone manifestations in children with pre-B ALL, and the reported findings unravel the mechanisms of leukemia-induced bone loss and provide evidence that zoledronic acid not only compensates for leukemia-dependent bone fragility but also reduces leukemia burden. Zoledronic acid, a bisphosphonate, is the current standard of care for treating bone metastases, in conjunction with standard antineoplastic therapy [64]. Additionally, zoledronic acid has been shown to be safe and tolerable when administered in combination with chemotherapy to children with ALL for treatment-related osteonecrosis [65]. In our study, zoledronic acid was administered when the disease burden was low in the bone marrow, highlighting its clinical potential in the setting of minimal disease, and to alleviate leukemia-induced bone fragility. Future studies to expand this paradigm into other genetic subtypes of pre-B ALL, and testing the efficacy of combining zoledronic acid with contemporary therapy are warranted. In addition, the role of zoledronic acid in glucocorticoid-induced bone loss needs to be further explored. Taken together, these findings provide strong rationale for targeting the BMM in BCR-ABL1^+^ pre-B ALL, with zoledronic acid identified as a potential therapeutic candidate for clinical application.

## Electronic supplementary material

Supplementary Figure Legends

Supplementary Figure 1

Supplementary Figure 2

Supplementary Figure 3

Supplementary Figure 4

Supplementary Figure 5

Supplementary Figure 6

Supplementary Figure 7
