# Supplementary material for: New therapeutic opportunities from dissecting the pre-B leukemia bone marrow microenvironment
Source: Leukemia. 2018 May 8;32(11):2326–38. doi: 10.1038/s41375-018-0144-7 (PMC6224400; doi:10.1038/s41375-018-0144-7)
Supplement: Supplementary file 1 — Supplementary Figure Legends [file 41375_2018_144_MOESM1_ESM.docx]

**Supplementary Figure 1. Homing and hematopoiesis in mice with pre-B leukemia.** (**A**) Flow cytometric profiles of the pre-B leukemia cells. (**B**) Percentage of mCherry^+^ cells in bone marrow and spleen 24 hours after injecting 1x10^6^ leukemia cells (*n* = 4 mice). (**C**) Total number of spleen cells during leukemia development. (**D**) Percentage of CD3^+^ cells in the CD45^+^mCherry^-^ fraction during leukemia development. (**E**) Percentage of myeloid subpopulations in the CD45^+^mCherry^-^ fraction during leukemia development. (**C to E**) Mice were injected with 1x10^3^ leukemia cells. Day 8, 10, 13, 15, and 16: *n* = 4 mice; Day 17: *n* = 3 mice; Day 20: *n* = 6 mice**.** * *P* < 0.05, ** *P* < 0.01, *** *P* < 0.001, **** *P* < 0.0001. Error bars represented mean ± SD.

**Supplementary Figure 2. Flow cytometric gating strategy for hematopoietic cells and bone marrow stromal cells.** (**A**) Subpopulations of CD45^+^ hematopoietic cells: NK cells (CD3^-^NK1.1^+^), CD3 T cells (CD3^+^NK1.1^-^), neutrophils (B220^-^CD11b^+^Ly6G^+^), macrophages (B220^-^CD11b^+^Ly6G^-^F4/80^+^), Ly6C^lo^ monocytes (B220^-^CD11b^+^Ly6G^-^Ly6C^lo^), Ly6C^hi^ monocytes (B220^-^CD11b^+^Ly6G^-^Ly6C^hi^), pro-B cells (B220^+^CD19^+^CD11b^-^CD43^+^IgM^-^), pre-B cells (B220^+^CD19^+^CD11b^-^CD43^-^IgM^-^), immature B cells (B220^+^CD19^+^CD11b^-^CD43^-^IgM^+^IgD^-^), and mature B cells (B220^+^CD19^+^CD11b^-^CD43^-^IgM^+^IgD^+^). (**B**) Subpopulations of CD45^-^Ter119^-^ bone marrow stromal cells: endothelial cells (CD31^+^), CXCL-12 abundant reticular (CAR) cells (Sca-1^-^CD31^-^PDGRFβ^+^), PDGFRα^+^Sca-1^+^CD31^-^ (PαS) mesenchymal cells, mesenchymal stem/stromal cells (MSCs) (Sca-1^+^CD31^-^CD51^+^), and osteoblastic cells (Sca-1^-^CD31^-^CD51^+^).

**Supplementary Figure 3. The composition of bone marrow stromal cells during leukemia development.** (**A**) Number of MSCs. (**B**) Number of PαS mesenchymal cells. (**C**) Number of CAR cells. (**A-C**) Bone marrow cells were harvested from one femur and two tibias after enzymatic dissociation (Day 8, 10, 13, 15, and 16: *n* = 4 mice; Day 17: *n* = 3 mice; Day 20: *n* = 6 mice)**.** (**D**) Number of vimentin+ fibroblasts. Bone marrow cells were harvested from both femurs of control mice (*n* = 4) and Day 16 leukemia mice (*n* = 4). Error bars represented mean ± SD.

**Supplementary Figure 4. Leukemia does not affect the cortical bone in the mid-femoral diaphysis.** Micro-CT analysis of the cortical bone in the mid-femoral diaphysis (*n* = 4 mice per time point). (**A**) Cortical volume (Ct.V). (**B**) Marrow volume (Ma.V). (**C**) Cortical thickness (Ct.Th). (**D**) Endosteal perimeter (En.Pm). (**E**) Periosteal perimeter (Ps.Pm). Error bars represented mean ± SD.

**Supplementary Figure 5. Histological analysis of the femoral bone sections during leukemia development.** (**A**) Number of TRAP^+^ osteoclasts per bone surface in the distal femur bone compartment. (**B**) Number of osteoblastic cells in the distal femur bone compartment. (**C**) Number of osteoblastic cells per bone surface in the distal femur bone compartment. Throughout, *n* = 4 mice per time point. * *P* < 0.05, ** *P* < 0.01, *** *P* < 0.001. Error bars represented mean ± SD.

**Supplementary Figure 6. Zoledronic acid rescues the bone loss during leukemogenesis.** Micro-CT analysis of femur trabecular bone in mice treated with zoledronic acid or vehicle at day 15 post leukemia cell injection ( *n* = 4). (**A**) Trabecular spacing (Tb.Sp). (**B**) Trabecular number (Tb.N). (**C**) Trabecular thickness (Tb.Th). ** *P* < 0.01, *** *P* < 0.001, **** *P* < 0.0001. Error bars represented mean ± SD.

**Supplementary Figure 7. Zoledronic acid does not alter homing of leukemia cells and disease manifestations when mice succumb to leukemia.** (**A**) Percentage of leukemia cells in the bone marrow and spleen of mice following treatment with either 4 doses of zoledronic acid or vehicle (*n* = 4). (**B**) Number of cells in the bone marrow and spleen when mice treated with either zoledronic acid or vehicle succumb to disease. (**C**) Percentage of leukemia cells in the bone marrow, spleen, and blood when mice treated with either zoledronic acid or vehicle succumb to disease. (**B-C**), *n* = 9 mice in vehicle treated group and *n* = 7 mice in zoledronic acid treated group. Error bars represented mean ± SD.
