# Supplementary figures and images for: New therapeutic opportunities from dissecting the pre-B leukemia bone marrow microenvironment
Source: Leukemia. 2018 May 8;32(11):2326–38. doi: 10.1038/s41375-018-0144-7 (PMC6224400; doi:10.1038/s41375-018-0144-7)

Supplementary Figure 1

A

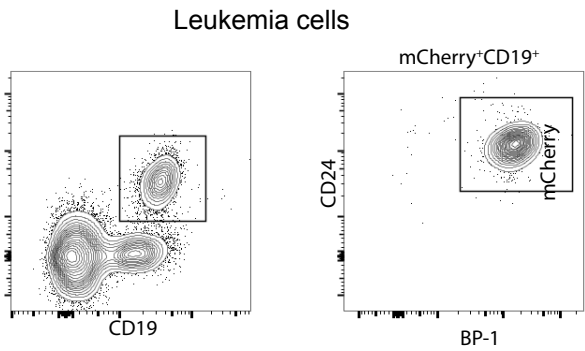

B

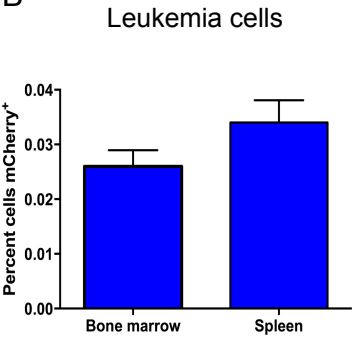

C

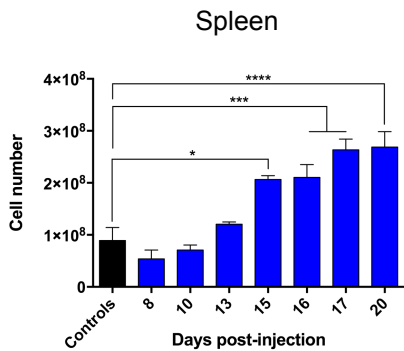

D

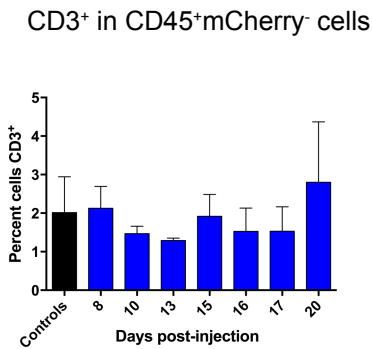

E

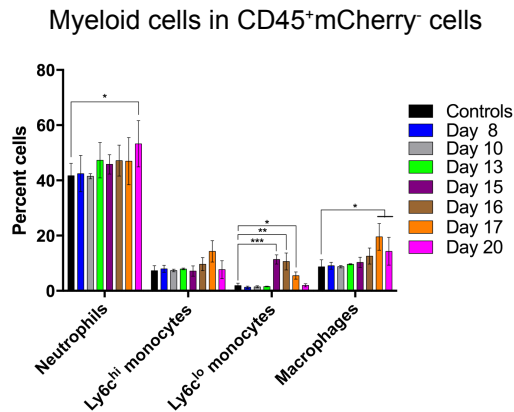

Supplement: Supplementary file 2 — Supplementary Figure 1 [file 41375_2018_144_MOESM2_ESM.pdf]

A

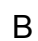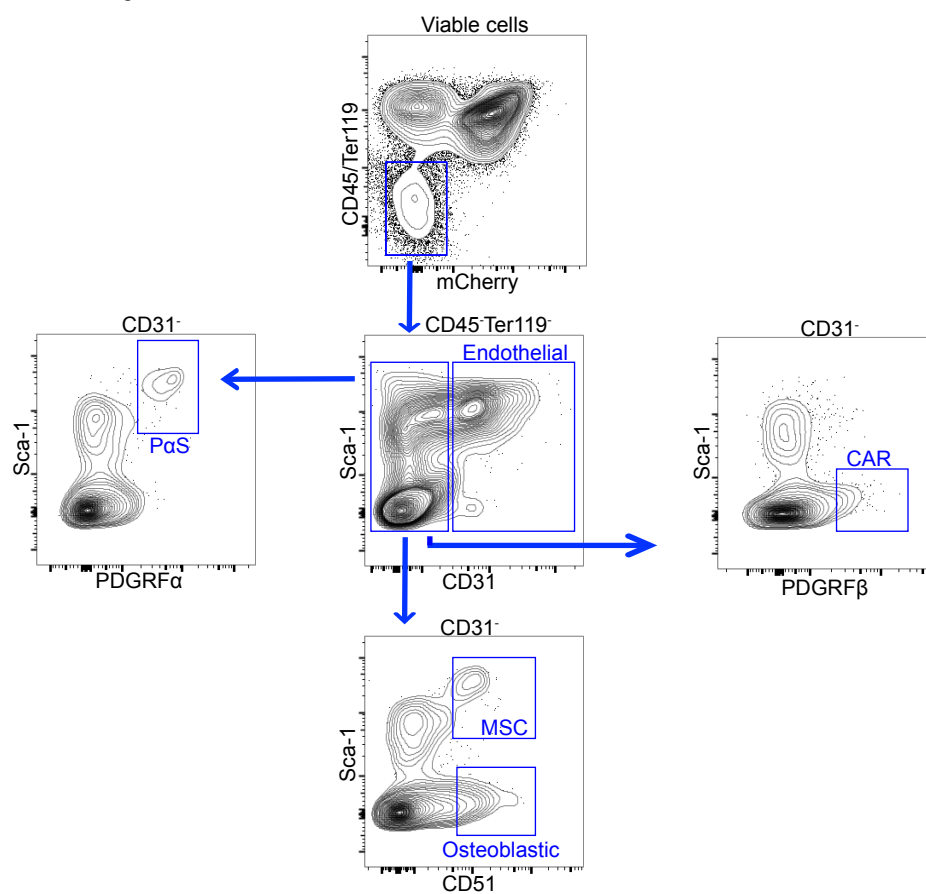

Supplement: Supplementary file 3 — Supplementary Figure 2 [file 41375_2018_144_MOESM3_ESM.pdf]

Supplementary Figure 3

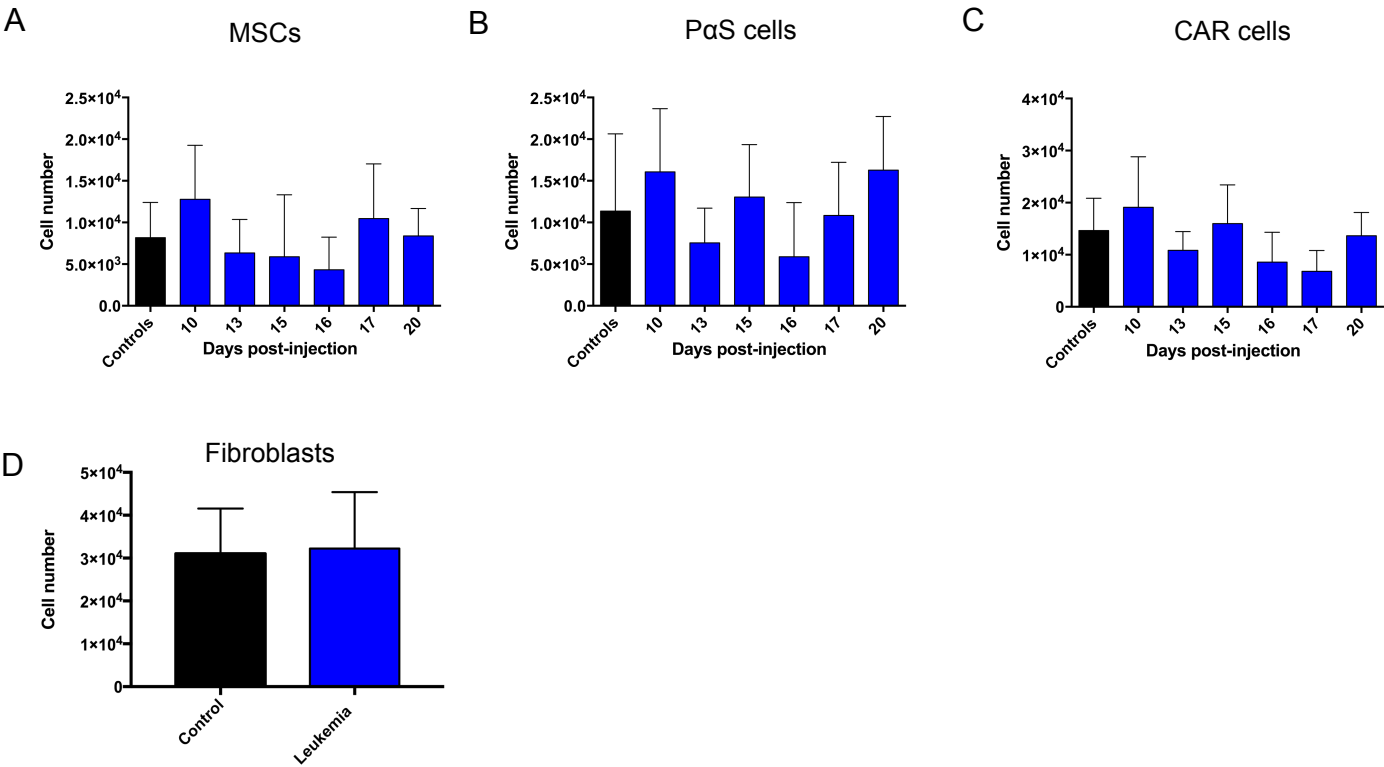

Supplement: Supplementary file 4 — Supplementary Figure 3 [file 41375_2018_144_MOESM4_ESM.pdf]

Supplementary Figure 4

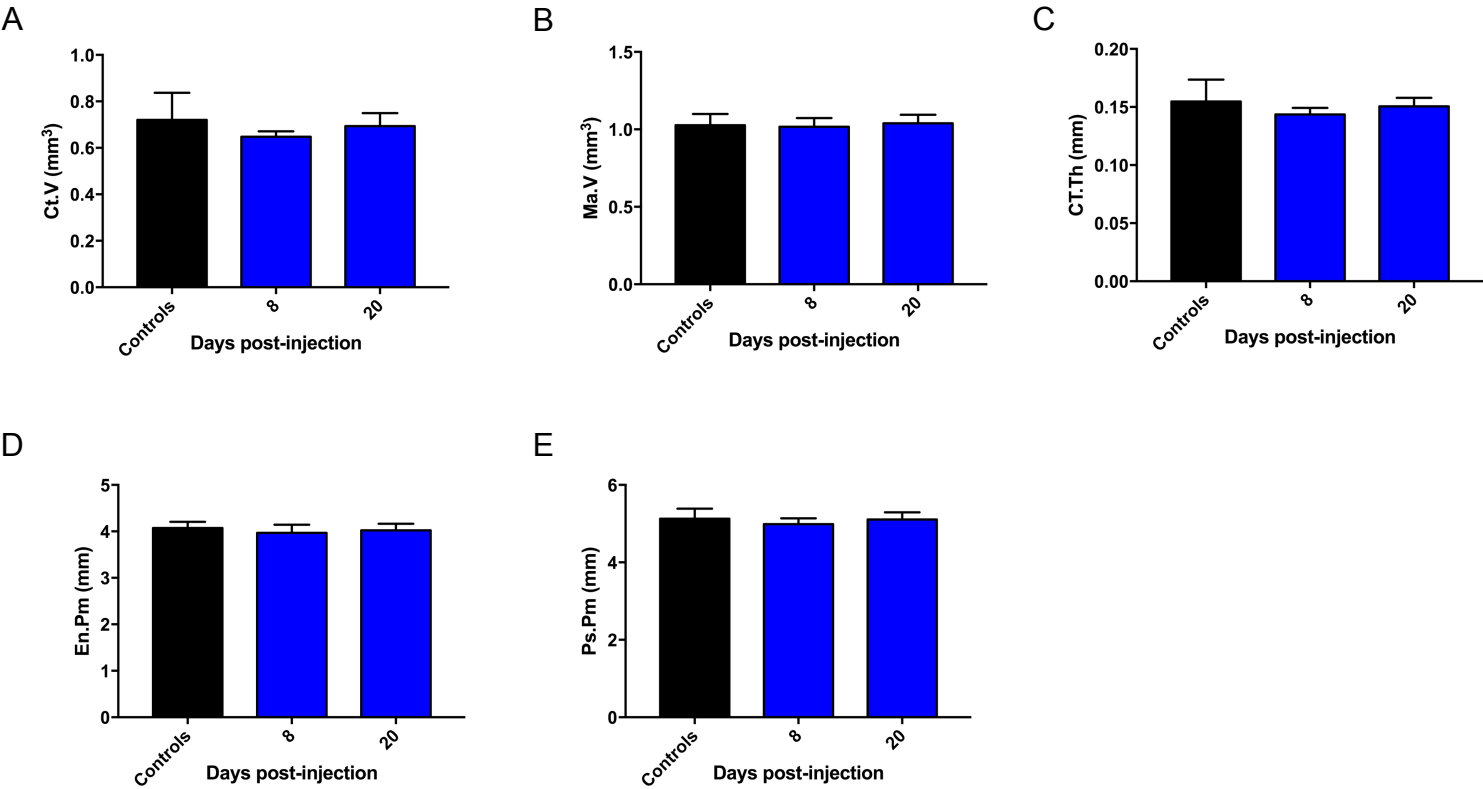

Supplement: Supplementary file 5 — Supplementary Figure 4 [file 41375_2018_144_MOESM5_ESM.pdf]

Supplementary Figure 5

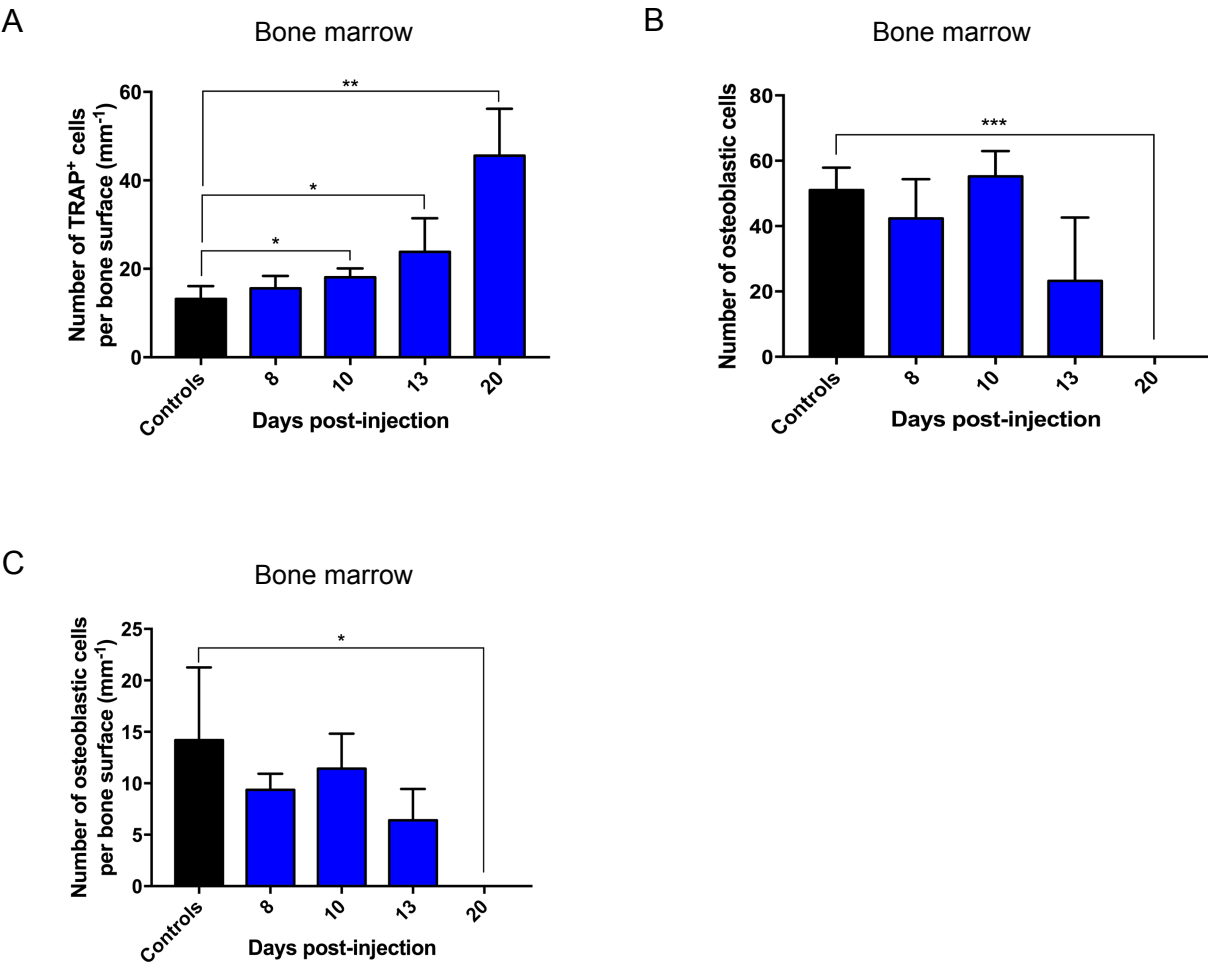

Supplement: Supplementary file 6 — Supplementary Figure 5 [file 41375_2018_144_MOESM6_ESM.pdf]

Supplementary Figure 6

A

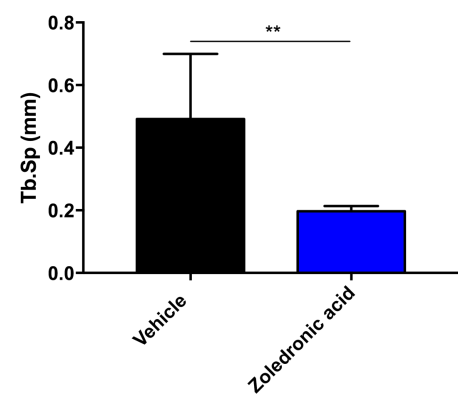

B

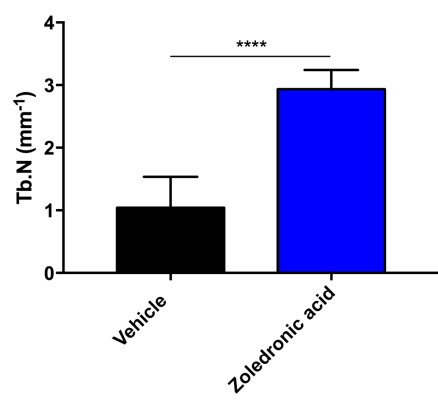

C

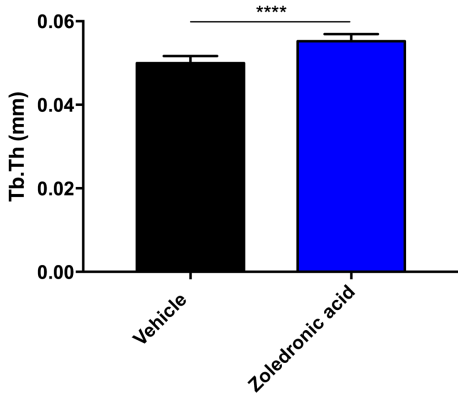

Supplement: Supplementary file 7 — Supplementary Figure 6 [file 41375_2018_144_MOESM7_ESM.pdf]

Supplementary Figure 7

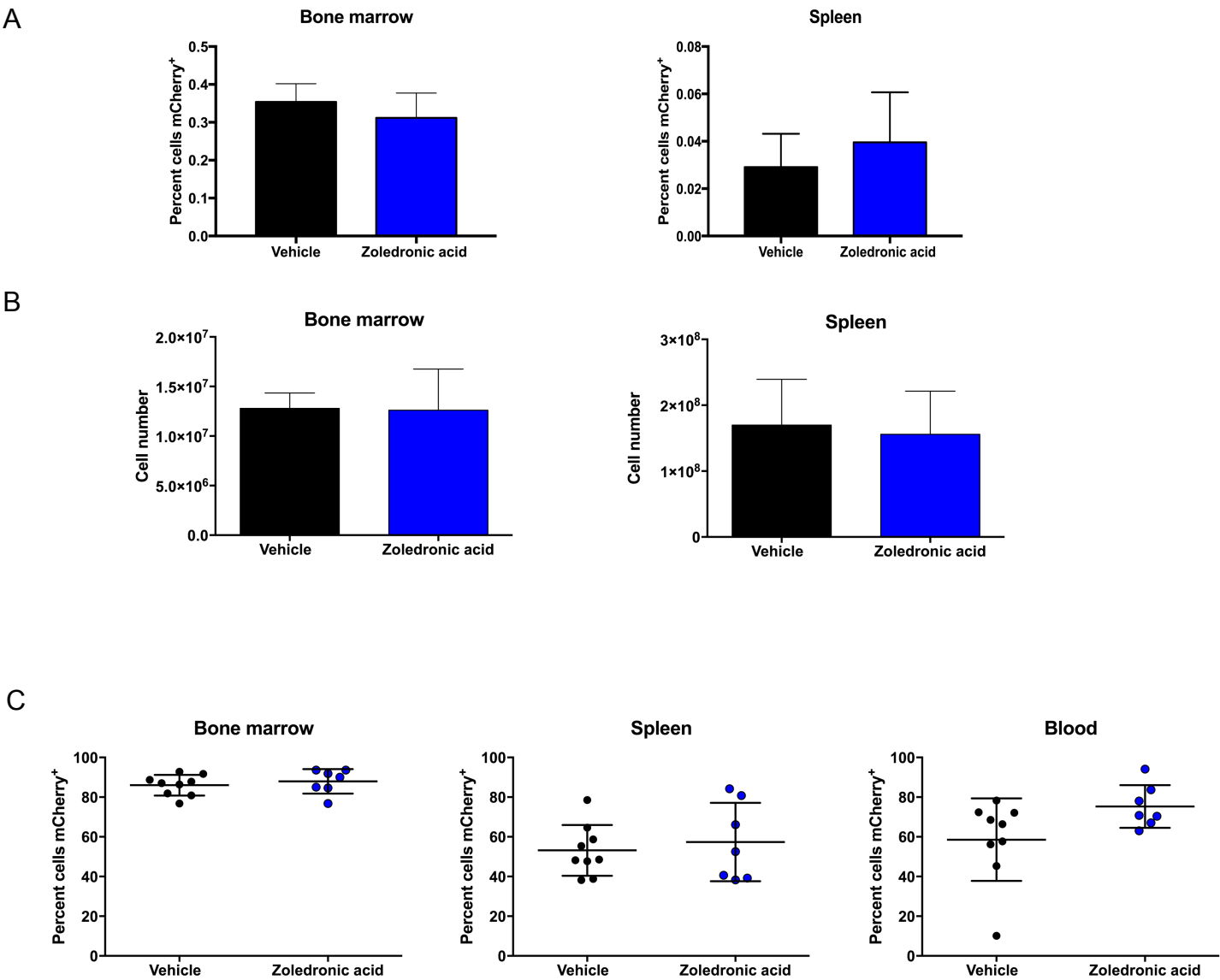

Supplement: Supplementary file 8 — Supplementary Figure 7 [file 41375_2018_144_MOESM8_ESM.pdf]
